# Exploring the Glycemic Control of Pay-for-Performance Program for Psychiatric Patients With Diabetes in Real World: A Retrospective Quasiexperimental Study

**DOI:** 10.1155/jdr/9660739

**Published:** 2025-06-04

**Authors:** Chin-Chou Yang, Wen-Chen Ouyang, Tsuo-Hung Lan, Yee-Yung Ng, Shiao-Chi Wu

**Affiliations:** ^1^Tsaotun Psychiatric Center, Ministry of Health and Welfare, Nantou, Taiwan; ^2^Institute of Health and Welfare Policy, National Yang Ming Chiao Tung University, Taipei, Taiwan; ^3^Department of Nursing, Hungkuang University, Taichung City, Taiwan; ^4^College of Agriculture and Health, Tunghai University, Taichung City, Taiwan; ^5^Department of Education and Research, Kai-Syuan Psychiatric Hospital, Kaohsiung City, Taiwan; ^6^Department of Nursing, Shu-Zen Junior College of Medicine and Management, Kaohsiung City, Taiwan; ^7^Department of Psychiatry, Medical College, Kaohsiung Medical University, Kaohsiung City, Taiwan; ^8^Department of Geriatric Psychiatry, Jianan Psychiatric Center, Ministry of Health and Welfare, Tainan City, Taiwan; ^9^Department of Medicine, National Yang Ming Chiao Tung University, Taipei, Taiwan; ^10^Center for Neuropsychiatric Research, National Health Research Institutes, Miaoli, Taiwan; ^11^Institute of Clinical Medicine, National Yang Ming Chiao Tung University, Taipei, Taiwan; ^12^Department of Medicine, Fu Jen Catholic University Hospital, Fu Jen Catholic University, New Taipei City, Taiwan; ^13^Department of Long-Term Care, College of Nursing, Asia University, Taichung, Taiwan

**Keywords:** diabetes care, health policy, mental illness, pay-for-performance

## Abstract

**Background:** Psychiatric patients with Type 2 diabetes often experience suboptimal care and poor health outcomes.

**Aims:** This study is aimed at investigating the impact of a diabetes pay-for-performance (P4P) program on glycemic control in psychiatric patients with diabetes by comparing two regional psychiatric hospitals, one with the P4P program and one without.

**Methods:** We conducted a retrospective quasiexperimental study. A total of 149 psychiatric outpatients with Type 2 diabetes were enrolled in the P4P group, and 129 patients were in the non-P4P group. Hemoglobin A1c (HbA1c) values in the fourth quarter of 2018 served as baseline (before P4P implementation in either hospital). Follow-up HbA1c levels were collected at 3, 6, 9, and 12 months in 2019. Propensity score matching was performed based on baseline HbA1c to create comparable groups. Changes in HbA1c over 1 year were analyzed using paired and independent *t*-tests and a generalized estimating equation (GEE) model.

**Result:** The mean HbA1c level in the P4P group decreased progressively over 12 months (from 6.97% at baseline to 6.60%), whereas the non-P4P group showed an increase (from 7.00% to 7.12%). By the fourth quarter, the P4P group had a significantly lower mean HbA1c than the non-P4P group (*p* < 0.05). Subgroup analysis showed a greater HbA1c reduction in P4P participants who were male or had schizophrenia (*p* = 0.01 and *p* = 0.04, respectively).

**Conclusions:** The P4P program was associated with significantly improved glycemic control in psychiatric patients with diabetes compared to usual care. This integrated care model may be an effective strategy to improve diabetes outcomes in psychiatric populations.

## 1. Introduction

Diabetes mellitus is a growing global health concern. The number of people with diabetes worldwide is projected to reach 642 million by 2040 [1]. The prevalence of diabetes is considerably higher among individuals with mental illness, especially those with schizophrenia [2, 3]. People with schizophrenia have a 2- to 5-fold greater risk of developing Type 2 diabetes than the general population [4]. Multiple factors contribute to this elevated risk, including obesity, hypertension, hyperlipidemia, use of second-generation antipsychotics, physical inactivity, unhealthy diet, and low socioeconomic status [5]. Moreover, the quality of diabetes care for patients with psychiatric disorders is often poorer than that for the general population [6], and these patients suffer higher rates of diabetes-related complications and mortality compared to patients without mental illness [7].

To reduce these disparities, the American Diabetes Association (ADA) emphasizes patient-centered diabetes care delivered through collaborative and multidisciplinary team approaches. Coordinated management of diabetes—integrating medical and psychosocial care—can empower patients and improve self-management, thereby enhancing the quality of diabetes care for those with mental illness.

**Figure 1:**
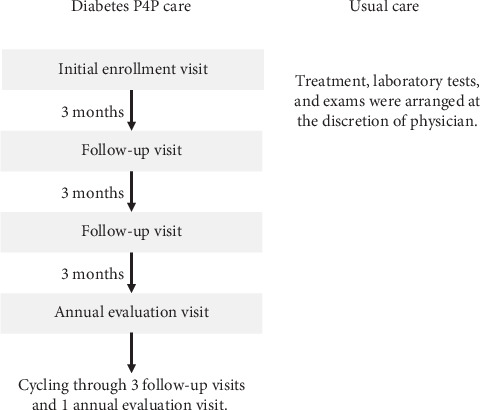
The flowchart of P4P care and usual care. The certain laboratory tests and exams were required in each visit in the P4P group but arranged at the discretion of the physician in the non-P4P (usual care) group. There are incentive fees for each visit of the P4P group, but no incentive fees for the non-P4P group.

Taiwan's National Health Insurance (NHI) program, which covers ~99.9% of the population since its establishment in 1995, introduced a P4P program for diabetes care in 2001 to improve care quality and prevent complications. The diabetes P4P program is a patient-centered, multidisciplinary team care model involving physicians, diabetes nurse educators, and dietitians. Patients receive structured diabetes care plans and diabetes self-management education (DSME) at enrollment, regular follow-up (typically every 3 months), and annual evaluations. Required laboratory tests (e.g., fasting glucose, hemoglobin A1c (HbA1c), lipids, renal function) are scheduled quarterly, and examinations for complications (e.g., urine albumin-to-creatinine ratio, blood pressure, eye fundus, and foot exams) are performed at least annually. In addition to standard reimbursement for services, healthcare providers receive incentive payments for each P4P patient at enrollment (TWD$650 ($23.36)), follow-up (TWD$200 ($7.19)), and annual review (TWD$800 ($28.75)), provided that all required indicators are documented and reported via a designated online system.  Figure 1 presents the P4P program flow, and Table 1 outlines differences between P4P and usual diabetes care requirements.

Psychiatric wards/units in general and psychiatric hospitals have existed in Taiwan for many years. Psychiatric hospitals are special systems in Taiwan that provide comprehensive psychiatric services (inpatient, outpatient, and emergency care). Most psychiatric hospitals are staffed by psychiatrists who treat both mental illness and general medical conditions, without on-site endocrinologists or other specialists. One regional psychiatric hospital in Taiwan hired an endocrinologist and joined the P4P program of diabetes care in January 2019, whereas other regional psychiatric hospitals continued to provide usual diabetes care without P4P during that period.

Evidence on the effectiveness of diabetes P4P programs in psychiatric populations is very limited. To our knowledge, no prior study in Asia has directly compared outcomes between psychiatric patients with diabetes receiving P4P care and those receiving standard care. Therefore, we conducted this retrospective quasiexperimental study to evaluate whether the P4P program improves glycemic control in psychiatric patients with Type 2 diabetes.

## 2. Methods

### 2.1. Study Design and Participants

This retrospective quasiexperimental study compared diabetes control in two regional psychiatric hospitals: one with the P4P program and one without the P4P program. We included all patients with a diagnosis of Type 2 diabetes mellitus who were treated in the outpatient departments of these two hospitals between January and December 2019. The hospital that joined the P4P program (the P4P hospital) had an endocrinologist-led diabetes care team in place starting in 2019. The other hospital (the non-P4P hospital) provided usual diabetes care through psychiatrists without a specialized diabetes team. At the P4P hospital, a total of 157 psychiatric outpatients with diabetes were identified in 2019; after excluding 8 patients (3 were hospitalized for acute psychiatric issues, 3 were lost to follow-up, and 2 died during the year), 149 patients remained in the P4P group. In the non-P4P hospital, 129 psychiatric outpatients with diabetes were identified and included for analysis, after excluding 5 patients (3 were hospitalized for acute psychiatric issues, and 2 were lost to follow-up). The two hospitals were comparable in size and services, each with over 500 psychiatric beds and providing inpatient, outpatient, emergency, and community psychiatric care. Both hospitals employed psychiatric mental health nurses and dietitians who were certified diabetes educators to support diabetes management for their patients.

### 2.2. P4P Program Versus Usual Care

Patients in the P4P hospital were referred to a coordinated multidisciplinary diabetes care team consisting of an endocrinologist, a diabetes nurse educator, and a dietitian. At enrollment, each P4P patient received a comprehensive assessment and an individualized diabetes care plan. The team reviewed the patient's medical and psychiatric status, discussed treatment goals, and agreed on management strategies in collaboration with the patient. Key components of care included regular DSME sessions (at enrollment and every 3 months during follow-up visits) emphasizing psychosocial empowerment and self-care skills. DSME covered nutritional counseling, physical activity, glucose self-monitoring, medication adherence, and lifestyle modifications (e.g., smoking cessation, moderation of alcohol use, and adequate sleep). Short-term and long-term diabetes management goals were set with each patient, and follow-up plans were arranged to reinforce adherence and provide ongoing support. At each follow-up (approximately quarterly) and annual evaluation visit, required laboratory tests were conducted (including HbA1c and fasting glucose, at least every 3 months, and lipid profile, liver, and renal function tests, etc., as per the standard P4P protocol). The P4P hospital received additional incentive payments for these services when all required clinical data were collected and reported to the NHI system on schedule. In contrast, patients at the non-P4P hospital received usual care for diabetes from their treating psychiatrists. There was no structured multidisciplinary intervention or scheduled DSME in the non-P4P setting; laboratory tests and diabetes education were provided at the discretion of the individual physician without the additional coordination or incentive-driven reporting that characterized the P4P program.

### 2.3. Data Collection and Outcome Measures

Baseline glycemic control was defined by the HbA1c value measured in the fourth quarter of 2018, before the implementation of the P4P program at the P4P hospital. We collected HbA1c values for each patient at approximately 3 months (first quarter 2019), 6 months (second quarter), 9 months (third quarter), and 12 months (fourth quarter) after the start of 2019 from the electronic medical record systems of both hospitals. The primary outcome was the change in HbA1c over the 1-year follow-up period. Patient characteristics recorded for analysis included age, sex, primary psychiatric diagnosis (with categories including schizophrenia, bipolar disorder, and dementia), and comorbid chronic conditions (hypertension and hyperlipidemia).

### 2.4. Statistical Analysis

Continuous and categorical variables for the two hospital groups were compared using independent-samples *t*-tests. To reduce baseline differences between groups, we performed propensity score matching [8] (1:1 nearest-neighbor without replacement) based on the baseline HbA1c level. This yielded two matched cohorts with similar baseline glycemic control for comparison. Postmatching group differences in mean HbA1c were assessed with independent *t*-tests at baseline and at 12 months, and within-group changes in HbA1c over time were assessed with paired *t*-tests.

We further evaluated the effect of the P4P intervention on HbA1c over time using generalized estimating equation (GEE) models for longitudinal data. GEE is appropriate for repeated-measures analysis as it accounts for within-subject correlations [9] and can provide robust estimates even when data are missing at random [10, 11]. We constructed a GEE model with HbA1c as the dependent variable, time (quarter) and group (P4P vs. non-P4P) as independent factors, and the interaction of group-by-time to estimate the differential change in HbA1c. An adjusted GEE model was also run including covariates (age, sex, major psychiatric diagnosis, hypertension, and hyperlipidemia) to control for potential confounders. All analyses were performed using IBM SPSS Statistics Version 26 (IBM Corp., Armonk, New York, United States). A two-sided *p* value < 0.05 was considered statistically significant. As this study included all eligible patients during the defined period, no a priori sample size calculation was required.

### 2.5. Ethical Considerations

This study was conducted in accordance with the Declaration of Helsinki. The research protocol was approved by the Institutional Review Board of Jianan Psychiatric Center, Ministry of Health and Welfare, Taiwan (IRB Approval Number: 20-011). The requirement for informed consent was waived due to the retrospective study design.

## 3. Results

The baseline characteristics of the psychiatric patients with diabetes in the P4P and non-P4P hospital groups are detailed in Table 2. Before the matching process, patients in the P4P group were significantly younger on average than those in the non-P4P group (*p* < 0.05). The mean baseline HbA1c was slightly lower in the P4P group (6.97% ± 1.20) compared to the non-P4P group (7.19% ± 1.60, *p* < 0.05). The P4P group had a higher prevalence of hypertension (*p* < 0.05) and a lower prevalence of dementia (*p* < 0.01) than the non-P4P group. After propensity score matching on baseline HbA1c, there were 114 patients in each group. The matched P4P and non-P4P cohorts were well balanced in baseline HbA1c and other characteristics (no significant differences in age, sex, or comorbidities).

Among the matched cohorts, baseline HbA1c levels stratified by sex, hypertension, hyperlipidemia, schizophrenia, bipolar disorder, or dementia did not differ significantly between the P4P and non-P4P groups. After 1 year of follow-up, the P4P group showed greater improvement in glycemic control. By the fourth quarter of 2019, the mean HbA1c in the P4P group was significantly lower than in the non-P4P group for male patients (*p* = 0.01) and for patients with schizophrenia (*p* = 0.04). Within-group analyses indicated that, after 1 year of the P4P program, there were significant reductions in HbA1c from baseline among P4P patients who were male, as well as those with hyperlipidemia, schizophrenia, or bipolar disorder (all *p* < 0.05). In contrast, the non-P4P group did not exhibit significant within-group HbA1c improvements in these subgroups over the year (Table 3).

Table 4 summarizes the trends in HbA1c over time and the factors associated with HbA1c levels. In the non-P4P group, mean HbA1c fluctuated throughout 2019: It decreased slightly by the third quarter (9 months) but rose again by Q4, ending slightly above the baseline level. In the P4P group, mean HbA1c declined steadily from the second quarter (6 months) through the end of the year. In the unadjusted (crude) GEE analysis, the P4P group had significantly lower mean HbA1c than the non-P4P group by Q3 (9 months) and Q4 (12 months) of 2019 (*p* < 0.05 for group difference). In the adjusted GEE model controlling for covariates, the mean HbA1c remained significantly lower in the P4P group at Q4 (interaction *p* < 0.05), confirming a beneficial effect of the P4P program on glycemic control relative to usual care. Additionally, in the GEE model, the presence of hyperlipidemia was associated with a higher overall HbA1c level (*β* > 0, *p* < 0.05), whereas a diagnosis of schizophrenia was associated with a lower HbA1c level (*β* < 0, *p* < 0.05), independent of the intervention.

## 4. Discussion

To the best of our knowledge, this is the first study in Asia with a comparison group to evaluate the benefits of a diabetes P4P program for psychiatric patients with diabetes. We found that after 1 year of implementation, patients in the P4P program achieved significantly better glycemic control than those receiving usual care. The mean HbA1c in the P4P group decreased from 6.97% at baseline to 6.60% at 12 months, while the non-P4P group's mean HbA1c increased from 7.00% to 7.12% over the same period. According to ADA standards of care, an HbA1c < 7% is a recommended target for many nonpregnant adults [12]. Our findings show that the P4P intervention helped more patients attain this glycemic goal, suggesting that such a program can be an effective strategy to improve diabetes outcomes in this high-risk population of psychiatric patients.

The improved outcomes observed in the P4P group can be attributed to the comprehensive, team-based approach inherent in the P4P program. With an integrated care team, patients likely received management that more closely adhered to clinical guidelines for diabetes. Regular consultations and psychosocial support through the P4P program may have enhanced continuity of care and treatment adherence [13–15]. Effective communication and collaboration between the diabetes care team and psychiatric patients are essential for successful diabetes management, as patients with mental illness often encounter barriers in accessing quality medical care. The P4P program's structure appears to have mitigated some of these barriers by actively engaging patients in care.

Another key component of the P4P program was the provision of DSME at routine intervals. DSME is a critical element of diabetes care that has been shown to significantly reduce HbA1c and improve psychosocial outcomes in general diabetic populations [16]. It can lead to healthier lifestyle behaviors, better quality of life, and reduced diabetes-related distress and depression [17]. However, patients with psychiatric disorders have often been excluded from clinical trials of DSME and other diabetes interventions [18], leaving uncertainty about the effectiveness of such education in this group. In our study, P4P participants received structured DSME every 3 months, whereas those in the non-P4P group did not. This regular, structured education likely played a pivotal role in the P4P group's significant improvement in HbA1c relative to the non-P4P group.

Notably, we observed that it took nearly a year for the full benefits of the P4P program to manifest in significantly lower HbA1c compared to usual care. Psychiatric patients may require a longer period to achieve glycemic improvement than the general diabetes population [19]. Several factors could contribute to this delay. First, many antipsychotic medications are associated with weight gain and adverse metabolic effects, which can complicate glycemic control [20]. Second, individuals with mental illness may face social and cognitive challenges that impede learning and adopting new health behaviors, thereby slowing improvements in diabetes outcomes. A previous study has suggested that comprehensive diabetes care plans for patients with mental illness, including educational, nutritional, and exercise components, may take longer to yield results [21]. These considerations help explain why significant HbA1c reductions in the P4P group emerged later in the follow-up period.

We also found that male patients benefited more from the P4P program in terms of glycemic improvement than female patients. By study end, men in the P4P group had a greater HbA1c reduction and reached HbA1c < 7% earlier than women in the P4P group. Sex differences in diabetes outcomes have been reported in previous research [22, 23], potentially due to biological factors (such as hormonal differences affecting insulin sensitivity) or differences in health behaviors. Further investigation is warranted to understand the gender-specific responses to diabetes interventions in psychiatric patients, and targeted strategies may be needed to ensure female patients derive equal benefit from programs like P4P.

The success of the P4P program in improving glycemic control may also be influenced by its performance-based financial incentives. In this study, providers in the P4P hospital were required to deliver care according to defined quality indicators (such as scheduled tests and education sessions) in order to receive the incentive payments. This likely resulted in more complete follow-up and proactive management for P4P patients than for those receiving standard care, leading to better glucose control and the expectation of fewer complications over time. In fact, broader evaluations of diabetes P4P programs have demonstrated reductions in long-term complications. For example, participation in the P4P program has been associated with greater reductions in mortality rates, particularly among high-risk subgroups such as patients with disabilities [24], and with a lower risk of progression to end-stage renal disease requiring dialysis [25]. These findings underscore the potential of the P4P approach to deliver meaningful clinical benefits and support its adoption in settings serving patients with mental illness.

### 4.1. Limitations

Our study has several limitations. First, we did not directly assess patients' adherence to lifestyle recommendations (e.g., dietary habits and physical activity) or the severity of psychiatric symptoms, both of which could potentially affect glycemic outcomes. Second, although we assumed that all physicians adhered to standard diabetes care guidelines, unmeasured differences in clinical practice between the two hospitals cannot be entirely excluded. Nevertheless, the application of propensity score matching helped to reduce baseline differences and confounding, thereby enhancing the internal validity of our comparison. Third, while improved glycemic control is generally associated with a reduced risk of diabetes-related complications and hospitalizations, our 1-year observation period was insufficient to assess long-term outcomes. Fourth, we did not perform a sample size calculation. However, because this study included all eligible patients during the defined period, a priori sample size estimation was not required. Finally, further multicenter studies with more diverse populations and extended follow-up durations are warranted to confirm the reproducibility and external validity of these results.

## 5. Conclusion

In conclusion, implementing a diabetes P4P program in a psychiatric hospital setting significantly improved glycemic control in patients with Type 2 diabetes compared to standard care. This study demonstrates that an integrated, incentive-driven care model can help bridge the quality gap in diabetes management for patients with mental illness. Wider adoption of such programs may improve health outcomes and reduce the long-term complications of diabetes in this vulnerable population.

## Figures and Tables

**Table 1:** Comparisons of P4P care and non-P4P care of diabetes.

**Items**	**Diabetes P4P care**	**Non-P4P care**
Patient-centered team care	Patients were treated by a multidisciplinary team including physicians, nurses, and nutritionists, who are certified as diabetes educators.	Patients are treated with a physician, who was not necessary to be a diabetes educator.

Initial enrollment visit	Laboratory tests: ✓ Fasting blood sugar ✓ HbA1C ✓ Lipid profile: T-Chol, LDL, TG, and HDL ✓ Serum creatinine and GPT ✓ ACR Exams: ✓ Blood pressure ✓ Eye fundus examination ✓ Foot examination	The tests, exams, and DSME were arranged at the discretion of physician.
Follow-up visit	Laboratory tests: ✓ Fasting blood sugar ✓ HbA1C	
Annual evaluation visit	Laboratory tests: ✓ Fasting blood sugar ✓ HbA1C ✓ Lipid profile: T-Chol, LDL, TG, and HDL ✓ Serum creatinine and GPT ✓ ACR Exams: ✓ Blood pressure ✓ Eye fundus examination ✓ Foot examination	

DSME	Performed in each visit: ✓ Self-management training ✓ Nutritional therapy ✓ Physical activity ✓ Diabetes complications ✓ Prevention of smoking ✓ Limiting alcohol consumption	DSME was not required.

*Note:* HbA1C: glycated hemoglobin; T-Chol: total cholesterol; ACR: urine albumin/creatinine ratio.

Abbreviations: DSME: diabetes self-management education; HDL: high-density lipoprotein; LDL: low-density lipoprotein; TG: triglyceride.

**Table 2:** Basic characteristics of psychiatric patients with diabetes.

**Variables**	**Before matching**			**After matching**		
	**P4P group (** **n** = 149**)**	**Non-P4P group (** **n** = 129**)**	**p** ** value**	**P4P group (** **n** = 114**)**	**Non-P4P group (** **n** = 114**)**	**p** ** value**
Gender						
Male	53.5%	53.0%	0.94	52.6%	53.5%	0.90
Age (years)	55.08 ± 13.2	58.3 ± 14.7	**0.04**	55.58 ± 13.20	58.04 ± 13.88	0.17
HbA1c baseline	6.8 ± 1.3	7.3 ± 1.7	**0.01**	6.97 ± 1.30	7.00 ± 1.29	0.86
Schizophrenia	52.3%	58.9%	0.11	56.1%	54.4%	0.79
Bipolar	10.7%	14.7%	0.93	14.0%	14.0%	1.00
Dementia	8.7%	27.1%	**< 0.01**	11.4%	20.2%	0.07
Hypertension	51.2%	35.6%	**0.01**	52.6%	58.7%	0.11
Hyperlipidemia	79.8%	71.1%	0.09	80.7%	77.2%	0.52

*Note:* Age and HbA1c baseline were presented as mean ± SD. The bold entries in the table indicate statistically significant results with *p* values less than 0.05.

**Table 3:** The HbA1c level (mean ± SD) between P4P and non-P4P groups at baseline and after 12-month P4P implementation.

**Variables**	**Baseline**			**12-month P4P implementation**			**Difference within groups**			
	**P4P group (** **n** = 114**)**	**Non-P4P group (** **n** = 114**)**	^#^ **p** ** value**	**P4P group (** **n** = 110**)**	**Non-P4P group (** **n** = 84**)**	^#^ **p** ** value**	**P4P group (** **n** = 110**)**	⁣^∗^**p value**	**Non-P4P group (** **n** = 84**)**	⁣^∗^**p value**
Total	6.97 ± 1.30	7.00 ± 1.29	0.86	6.60 ± 1.25	7.12 ± 1.85	**0.02**	−0.32 ± 1.17	**0.01**	0.03 ± 1.53	0.88
Male	6.71 ± 0.96	6.98 ± 1.36	0.22	6.42 ± 1.00	7.20 ± 1.96	**0.01**	−0.29 ± 0.67	**<0.01**	0.14 ± 1.62	0.54
Female	7.27 ± 1.55	7.03 ± 1.22	0.39	6.80 ± 1.48	7.01 ± 1.72	0.55	−0.35 ± 1.57	0.11	−0.13 ± 1.41	0.57
Hypertension	7.00 ± 1.29	7.10 ± 1.18	0.68	6.80 ± 1.42	7.47 ± 2.33	0.09	−0.20 ± 1.41	0.23	0.26 ± 1.46	0.33
Hyperlipidemia	7.09 ± 1.34	7.06 ± 1.31	0.90	6.71 ± 1.25	7.12 ± 1.91	0.11	−0.32 ± 1.23	**0.02**	−0.02 ± 1.63	0.93
Schizophrenia	6.79 ± 1.35	6.88 ± 1.15	0.68	6.32 ± 1.21	6.88 ± 1.55	**0.04**	−0.36 ± 1.20	**0.02**	−0.05 ± 1.26	0.78
Bipolar	6.85 ± 0.88	7.07 ± 1.10	0.54	6.51 ± 0.80	7.02 ± 1.56	0.28	−0.33 ± 0.50	**0.02**	0.09 ± 1.55	0.85

*Note:* The bold entries in the table indicate statistically significant results with *p* values less than 0.05.

^#^ *p* value: independent *t* test.

⁣^∗^*p* value: paired *t* test.

**Table 4:** Factors associated with HbA1c levels of diabetic psychiatric patients (*N* = 228).

**Items**	**P4P group**	**Non-P4P group**	**Crude model**			**Adjusted model**		
	**HbA1c** **Mean (SD)**	**HbA1c** **Mean (SD)**	**β**	**s** **e**	**p**	**β**	**s** **e**	**p**
P4P			−0.19	0.16	0.24	−0.10	0.17	0.57
Follow-up								
Baseline (ref = 0)	6.97 (1.30)	7.00 (1.29)						
3 months	7.02 (1.56)	7.06 (1.68)	0.04	0.07	0.56	0.01	0.11	0.96
6 months	6.75 (1.32)	7.03 (1.68)	−0.12	0.08	0.14	0.01	0.11	0.95
9 months	6.66 (1.17)	6.84 (1.60)	−0.24	0.08	**< 0.01**	−0.19	0.11	0.28
12 months	6.60 (1.25)	7.12 (1.85)	−0.21	0.10	**0.03**	0.02	0.15	0.91
P4P^a^ follow-up								
3 months			0.02	0.19	0.86	0.05	0.15	0.74
6 months			−0.25	0.17	0.90	−0.23	0.16	0.15
9 months			−0.34	0.16	**0.04**	−0.12	0.15	0.43
12 months			−0.42	0.17	**0.01**	−0.41	0.19	**0.04**
Age			−0.01	0.01	0.22	−0.02	0.01	**0.01**
Gender (ref = female)			−0.19	0.17	0.27	−0.25	0.15	0.10
Hypertension (ref = 0)			0.21	0.17	0.23	0.31	0.16	0.06
Hyperlipidemia (ref = 0)			0.51	0.19	**0.01**	0.50	0.18	**< 0.01**
Schizophrenia (ref = 0)			−0.36	0.17	**0.04**	−0.66	0.26	**0.01**
Bipolar (ref = 0)			−0.04	0.19	0.83	−0.46	0.27	0.09
Dementia (ref = 0)			0.21	0.26	0.42	−0.17	0.34	0.63

*Note:* Using the generalized estimating equations (GEEs) to predict change in mean HbA1c level over 3, 6, 9, and 12 months from baseline. The bold entries in the table indicate statistically significant results with *p* values less than 0.05.

^a^The interaction between P4P and time (follow-up).

## Data Availability

Research data are not shared.
